# Two Minor Operations in Laryngology

**Published:** 1908-10-03

**Authors:** 


### The General Practitioner's Column.

[Contributions to this Column are invited, and if accepted will be paid for.]

### TWO MINOR OPERATIONS IN LARYNGOLOGY.

### Removal of Anenoids and Enlarged Tonsils.

With the advent of medical inspection of school children, this little operation will probably be practised more than ever, and rightly so, seeing that the evil results of nasal obstruction, especially in early life, seem the graver the more they are looked into. Happily, "there is no minor operation in the whole of surgery," says Parker, "which has more beneficial results." Failing special indications, choice of anæsthetic is settled chiefly by age, A.C.E. mixture being used up to twelve years old, and gas after then. In the first case, a nurse or other assistant is needed besides the operator and the anæsthetist. The little patient is laid supine on a table, the hair being covered by a waterproof cap; the head should neither be raised by a pillow nor dependent over the end of the table. The anæsthetist, of course, stands at the patient's head, and the surgeon at his right shoulder. Behind the surgeon is the nurse, who leans over the child controlling its hands. Between the surgeon and the anæsthetist is a large bowl placed on a stool below the edge of the table, and opposite the patient's head. While the brisk reflex still persists, a Doyen's gag is inserted between the incisors, and the mouth widely opened; the gag should be placed on the left side of the mouth and must be entirely to the left of the middle line, so as not to interfere with the free movement of the curette. With the child still on its back, a caged curette, held dagger fashion, is passed up behind the uvula until it impinges upon the posterior end of the septum, then swept firmly round the vault of the naso-pharynx and down again, pressure being maintained until the instrument is brought out of the mouth, in which case it usually brings away the growth. The correct swing of the curette is a veritable *tour de maître*, and requires some practice; the cutting-blade should describe a circular sweep from the top of the pharynx to the mouth, so as to shear clearly through the lower end of the growth, and to avoid leaving tags. The nurse now turns the child completely on to its right side, the blood escaping out of its mouth into the bowl below; and this must be done quickly and at once, whether the adenoid has been completely removed or not, for in the supine position blood and fragments of growth may readily enter the lungs. The curetting may now be repeated, but it is unnecessary to curette the lateral portions of the naso-pharynx, which is then explored digitally, to make sure that all is clear. The operator next passes his left forefinger on to the left tonsil, and with this as a guide, a plain guillotine tonsillotome is inserted, its fenestra engaging the tonsil. To effect this satisfactorily, the edge of the fenestra must be worked up from below, since tonsils enlarge chiefly downwards. Then, while the anæsthetist makes counterpressure behind the angle of the jaw, strong outward leverage is exerted until the tonsillotome lies almost transversely across the mouth, its thumb-piece being quite opposite the patient's right side. The left forefinger is now shifted to the screw of the instrument to steady it, and the blade driven home with the right thumb. This process is reversed for the remaining side, the gag removed, and the child's face being quickly cleaned with a wet sponge, it is carried off to bed head downwards.

With gas and a suitable chair (in the case of older patients), the services of a nurse may be dispensed with, as the movements can be controlled by straps. The procedure is just as described above, except that the gag is inserted before the anæsthetic is begun, and there is no turning of the patient. The tonsillotome is best eschewed, and reduction of the tonsils effected by punch forceps under eucaine in patients over twenty, or when there have been repeated attacks of tonsillitis, as in such cases local formation of fibrous tissue may lead to severe hæmorrhage after amputation. This complication is not at all common otherwise. In all cases, before leaving, one should depress the tongue and inspect the pharynx for dependent tags of lymphoid tissue, which, if present, should be removed with punch-forceps or scissors. Tags should not be pulled off; any attempt to do this is likely to strip down the mucous membrane from the pharynx and leave a painful raw surface. Education in nasal respiration is to be enjoined.

### Removal of Nasal Polypi.

Practically speaking, this follows on diagnosis almost as certainly as does the opening of an abscess. It may be contra-indicated in rare instances when the condition is but part of syphilitic or malignant disease, but in the ordinary run, and vast majority of cases, the treatment of nasal polypi is as definite as their appearance. For these reasons and others to be mentioned, it is surprising that general practitioners do not undertake this little operation oftener. True, frequent recurrence may point to underlying ethmoidal bone affection, or a nasal discharge raise the suspicion of sinus disease, both conditions requiring a specialist's care; nevertheless, removal of polypi is nearly always the first step, no matter who the surgeon may be. In the laryngological clinic, this small task is often given to the tyro, who finds, too, no difficulty about diagnosis. Lastly, a patient who has been relieved of nasal obstruction from this cause is generally very grateful. Nasal polypi, bluish-white gelatinous looking bodies, which dimple on being touched with a probe and are freely movable, hang down from the roof of the nose, and from the middle turbinate, presenting a characteristic contrast to the surrounding red mucosa. For their removal, a nasal speculum, angular nasal dressing forceps, and a snare are necessary. A wisp of cotton wool, about one and a half inches by an inch, is soaked in 10 per cent. cocaine solution, then wrung out, and under reflected light, placed edgewise at the upper and back part of the nose, so that the cocaine may come into contact with the attachments of the polypi. The patient is cautioned not to swallow any that may run into his mouth. After eight minutes the pledget of cotton wool is withdrawn, and the snare inserted with its loop (about one and a half inches long by half an inch wide), parallel to the septum. The upper half of the loop should be passed up between the septum and the polypus, and the snare then so rotated that the lower part of the wire passes round the nearest polypus. The wire is advanced upwards and slightly outwards, so as to get well up to the attachments, the loop tightened, and its contents removed with a smart jerk. If the loop be forcibly tightened the pedicle will be cut through, but it is far better to complete the removal by traction, as in this way the attachment of the polypus is more thoroughly removed, and less bleeding results. For this reason, snares, such as Krause's, with an undivided barrel, into which the wire can be completely withdrawn, are not to be recommended, for they cut the polypus, and do not allow it to be dragged from its attachments. The snaring is repeated till all polypi in view are removed, bleeding being mopped up with little wads of dry wool. It is not well to do more than one side at a sitting. If the posterior nares are blocked, gas is necessary. The patient's mouth is held open with a Doyen's gag, the left forefinger passed up into the post-nasal space, and the attachments of the polypi defined. Long polypus forceps are passed through the nose, their points felt behind by the finger, and thus guided, made to grasp the pedicle of the growth high up near the roof of the post-nasal space. It is then a simple matter to twist the growth off and to bring it away by the nostril. In all cases, hæmorrhage quickly ceases when the face is sponged with cold water, but one should keep the patient under observation for twenty minutes or so. The only after treatment necessary is to stop with cotton wool, for twenty-four hours, the nostril on the side treated.

In the worst cases of nasal polypi, they grow so rapidly, that they cannot be kept clear by snaring. In such extensive ethmoidal disease is usually present, and other sinuses are frequently involved. A major operation under general anæsthesia is then indicated, curetting away diseased parts and opening and draining the affected sinuses.

